# Fidelity of implementation: development and testing of a measure

**DOI:** 10.1186/1748-5908-5-99

**Published:** 2010-12-30

**Authors:** Rosalind E Keith, Faith P Hopp, Usha Subramanian, Wyndy Wiitala, Julie C Lowery

**Affiliations:** 1 HSR&D Center for Clinical Management Research, VA Ann Arbor Health Care System (11H), Ann Arbor, MI, USA; 2 School of Social Work, Wayne State University, Detroit, MI, USA; 3 Richard L. Roudebush VA Medical Center, Indianapolis, IN, USA

## Abstract

**Background:**

Along with the increasing prevalence of chronic illness has been an increase in interventions, such as nurse case management programs, to improve outcomes for patients with chronic illness. Evidence supports the effectiveness of such interventions in reducing patient morbidity, mortality, and resource utilization, but other studies have produced equivocal results. Often, little is known about how implementation of an intervention actually occurs in clinical practice. While studies often assume that interventions are used in clinical practice exactly as originally designed, this may not be the case. Thus, fidelity of an intervention's implementation reflects how an intervention is, or is not, used in clinical practice and is an important factor in understanding intervention effectiveness and in replicating the intervention in dissemination efforts. The purpose of this paper is to contribute to the understanding of implementation science by (a) proposing a methodology for measuring fidelity of implementation (FOI) and (b) testing the measure by examining the association between FOI and intervention effectiveness.

**Methods:**

We define and measure FOI based on organizational members' level of commitment to using the distinct components that make up an intervention as they were designed. Semistructured interviews were conducted among 18 organizational members in four medical centers, and the interviews were analyzed qualitatively to assess three dimensions of commitment to use--satisfaction, consistency, and quality--and to develop an overall rating of FOI. Mixed methods were used to explore the association between FOI and intervention effectiveness (inpatient resource utilization and mortality).

**Results:**

Predictive validity of the FOI measure was supported based on the statistical significance of FOI as a predictor of intervention effectiveness. The strongest relationship between FOI and intervention effectiveness was found when an alternative measure of FOI was utilized based on individual intervention components that had the greatest variation across medical centers.

**Conclusions:**

In addition to contextual factors, implementation research needs to consider FOI as an important factor in influencing intervention effectiveness. Our proposed methodology offers a systematic means for understanding organizational members' use of distinct intervention components, assessing the reasons for variation in use across components and organizations, and evaluating the impact of FOI on intervention effectiveness.

## Background

When introduced into clinical practice, evidence-based interventions sometimes improve expected outcomes, but often fail. Much of the research examining implementation of interventions into clinical practice focuses on the multitude of contextual factors antecedent to implementation (*e.g. *leadership engagement, culture, and slack resources), as well as on the nature of the intervention itself (*e.g. *complexity, compatibility, relative advantage) [1]. There is also evidence of a relationship between the degree to which an intervention (complex or otherwise) is successfully implemented into an organization and patient outcomes [2], supporting the proposition that the fidelity with which an intervention is implemented mediates the relationship between contextual antecedents and the intervention's effectiveness. Intervention effectiveness is defined as the patient and organizational outcomes expected to be associated with the intervention. Empirical research to further explore the fidelity with which an intervention is implemented is necessary in order to realize those benefits of healthcare interventions.

Taking into account the role of fidelity of implementation (FOI) as a mediating variable between context and intervention effectiveness brings into question the assumption that once an intervention is introduced into an organization, it is used as intended, and acknowledges that the real world clinical environment is more susceptible to contextual factors than is the controlled research environment in which interventions are often designed. Consequently, a failure to achieve expected outcomes may be due to insufficient fidelity to the intervention rather than the inadequacy of the intervention itself. Examining FOI can help explain why expected outcomes were or were not achieved with the introduction of an intervention into practice [3].

The primary objective of this paper is to contribute to the understanding of FOI by (a) proposing a methodology for measuring FOI and (b) examining the association between FOI and intervention effectiveness (measured as inpatient resource utilization and mortality, controlling for patient characteristics that might influence these outcomes). The intervention in this study is a nurse practitioner (NP) case management program for patients with chronic heart failure, designed to improve the cardiac care of patients diagnosed with CHF (CHF) [4]. The key components of the intervention were the availability of an NP case manager and the collaboration the NP case managers and primary care providers (PCPs).

The following research questions were examined:

1. What is the FOI for each intervention component in each medical center?

2. What is the FOI of the intervention as a whole in each medical center?

3. How is FOI associated with intervention effectiveness?

### Conceptual framework: fidelity of implementation

The theoretical foundations of the proposed conceptual framework are drawn from the innovation implementation literature and the program evaluation literature. Theoretically, implementation has been defined as "the transition period during which targeted organizational members ideally become increasingly skillful, consistent, and committed in their use of an innovation" and FOI has been defined as "the consistency and quality of targeted organizational members' use of the specific innovation" [5]. The program evaluation literature defines FOI as "the determination of how well an intervention is implemented in comparison with the original program design during an efficacy and/or effectiveness study" [6].

**Figure 1:**
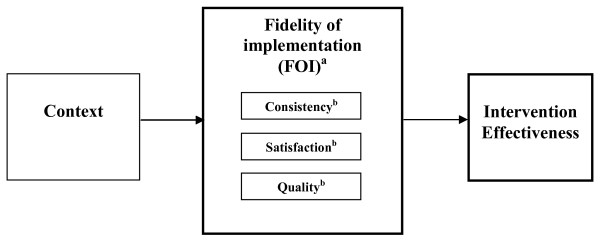
**fidelity of implementation**. ^a^FOI is assessed at the organizational level; ^b^consistency, satisfaction, and quality are measured at the organizational member level as dimensions of commitment to use.

We draw on elements of the preceding conceptualizations--the aspects of individual-level behavior (*i.e.*, organizational members' committed use) from the former and the aspects of achieving the intended program design from the latter (i.e. organizational members' actual use versus ideal use of the content of the intervention)--and put forth a conceptualization of FOI based on organizational members' level of commitment to using the distinct components of the intervention as they were designed, in order for the organization to achieve intended goals. We conceptualize commitment to use to be associated with an organizational member's personal acceptance and use of the innovation [5]. We investigated the following dimensions of commitment to use: satisfaction, quality, and consistency. *Satisfaction *represents organizational members' expressed level of enthusiasm with using the distinct components of the intervention, *quality *represents organizational members' expressed level of competence and knowledge regarding the use of the distinct components of the intervention, and *consistency *represents the frequency with which organizational members used the intervention based on program guidelines. Our overarching hypothesis is that FOI, as conceptualized in our model, will have a direct influence on intervention effectiveness (Figure 1).

### Clinical context: chronic heart failure

CHF is a leading cause of death in the United States, with significant morbidity, mortality, and healthcare costs; and the incidence and prevalence of the disease continue to increase [7]. CHF is a complex and debilitating disorder that seldom occurs as an isolated disease process. Patients with CHF tend to be older and more frail than the average patient; they also have more comorbidities and greater prevalence of confounding psychosocial, behavioral, and financial issues that can impede effective management of the CHF condition [8]. Currently, there is no cure for CHF; once diagnosed, ongoing treatment with medication is necessary for a person's remaining years of life [7]. Optimal treatment for CHF requires a holistic approach tailored to each patient's individual needs, mandating the use of a multidisciplinary approach to providing individualized care in order to maximize health outcomes [8].

In the past several years, nurse case management programs have been widely advocated as an effective multidisciplinary approach to the care of patients with CHF [8-10]. Nurse case management programs involve the provision of both medical and nonmedical care in order to address the range of patient needs, including a treatment plan that follows evidence-based guidelines and education and support for diet and medication compliance, or other unique patient circumstances. Several critical review papers evaluating the CHF disease management literature have concluded that case management programs reduce patient mortality rates, hospital admissions, length of stay, and hospital costs and improve patient functional status, quality of life, and compliance with care recommendations [8-11]. However, most of these studies were carried out using single-site designs with relatively homogenous patient samples. The studies that used multiple sites and a heterogeneous patient sample found that case management models do not always lead to an increase in beneficial patient outcomes [12-14]. Findings such as these demonstrate the uncertain association between the intervention and its effectiveness and the potential for the mediating effects of FOI.

## Methods

### Research design

The research presented in this paper is part of a larger implementation study that used a quasi-experimental, comparison-group design combined with qualitative interviews to investigate four Veterans Affairs (VA) medical centers' implementation of a CHF NP case management program [4]. The leadership at each of the medical centers hired one full-time cardiology NP case manager, with CHF case management as one of their responsibilities. One of the medical centers served as the referral center, with two full-time NPs to provide CHF case management, as well as offer consultative assistance to the NPs at the other three referring centers. With the exception of one medical center (medical center A), all of the centers were exposed to the intervention for four years. Medical center A, the referral center, had implemented the intervention about a year earlier than the other centers. Qualitative data for the present study are based on information collected from each medical center after the first 18 months of participation and focus on the perceptions of medical center staff concerning the intervention. We also report on quantitative outcomes collected at baseline and at one year after each patient's enrollment in the program.

For the study described in this paper, a mixed-methods sequential exploratory design was used to investigate implementation of the intervention at the four medical centers. Qualitative methods were first used to sufficiently explore FOI and to derive a measure of FOI. Quantitative methods were then used to compare patient outcomes (mortality and inpatient resource utilization) across the four medical centers. The rationale for selecting the two-phase mixed-methods sequential exploratory design was to use the quantitative patient outcomes to evaluate the predictive validity of the qualitatively derived measure of FOI [15,16]. The findings from the two phases were integrated by transforming the qualitative data into quantitative ratings of FOI in order to test the association between the FOI measure and intervention effectiveness [15].

### Setting

The unit of analysis for this study is the medical center, since we are trying to understand the determinants of the differences in intervention effectiveness at the organizational level. Two of the four participating medical centers were tertiary care centers (*i.e.*, large volume of patients, with specialists and teaching and research programs), and two medical centers were primary care centers (*i.e.*, few specialists, little or no teaching and research). Despite these differences, as part of the same integrated health system, many contextual features were very similar among the four medical centers, such as a standardized electronic medical record, thus limiting confounding of the outcomes by noncomparable contexts [17].

### Measures

#### Independent variables

FOI is the primary independent variable of interest. We measured FOI using qualitative methods to describe and rate the levels of satisfaction, quality, and consistency with which organizational members' committed to using the individual intervention components. Organizational members' commitment to use was rated on a scale consisting of five categories: 1 = nonuse, 2 = low compliance, 3 = compliant use, 4 = high compliance, and 5 = committed use [18]. FOI was first assessed for each intervention component at the organizational member level, then aggregated to produce an overall medical center rating of FOI for each individual component across members, and finally aggregated to produce an overall medical center rating of FOI.

Additional independent variables were included to control for patient characteristics that might be associated with the dependent variables, including age, race, number of comorbidities, medical center type (primary vs. tertiary), and the baseline values of the outcome variables one year prior to patient enrollment in the study.

#### Dependent variables

Intervention effectiveness is the outcome of interest in this study. Intervention effectiveness was measured by patient mortality and inpatient resource utilization. The latter was included as a measure of intervention effectiveness in addition to mortality because a major goal of the NP case management program was to help CHF patients avoid hospital admissions through optimal management of their CHF. The following measures of inpatient resource utilization were examined: number of hospital admissions with CHF as the primary reason for admission, number of all-cause hospital admissions, CHF hospital bed days of care, and all-cause hospital bed days of care.

#### Data collection

Qualitative data were collected through semistructured interviews with a total of 18 clinicians during the implementation of the NP case management program. A purposeful sampling strategy was used to select participants from each of the four medical centers for the interviews. Participants were selected based on their position in the medical center, to obtain perspectives from the different organizational members involved in the implementation of the case management program. Participants included a cardiologist, two to three primary care providers, and the NP case manager at each site. All participants gave informed consent, as approved by each medical center's Institutional Review Board. The interview protocol asked participants about perceptions and satisfaction related to different aspects of the case management program. Interviews were conducted over the phone. Data collection for the analyses reported in this paper took place from May 2004 through September 2004; this time period occurred approximately midway (18 months) into program implementation. The interviews were audio recorded and transcribed verbatim into Microsoft Word documents. Quantitative data on mortality and inpatient resource utilization were collected from national VA databases on participating patients for the year following their enrollment in the NP case management program. The total sample size for CHF patients enrolled in the study was 457.

### Data analysis: qualitative

#### Identification of components

The first step in the data analysis process involved delineating the eight distinct components of the CHF NP case management program based on the original grant submitted for the larger implementation study, in which each of the individual components was described with respect to the overall case management program:

1. Availability of an NP case manager: The NP case managers played a key role in (a) managing CHF patients' cardiac care and medical needs, (b) educating patients, (c) coordinating the care of patients with their PCP and with the inpatient referral center, and (d) providing onsite expertise to assist PCPs in the management of their CHF patients.

2. Collaboration between PCPs and NP case managers: A key element of the NP case management model was the collaborative relationship between the NP case manager and the patient's PCP. The NP case manager served to integrate subspecialty CHF care with primary care. Patient referrals from the PCP to the NP case manager of high-risk cardiac patients were essential to the success of the program. Additionally, successful collaboration between the PCP and the NP involved ongoing consultation and communication regarding patient care.

3. Coordination between primary care (referring) centers and inpatient (referral) center: The NP case manager was responsible for coordinating the hospitalization and discharge planning of CHF patients with the referral center.

4. Provision of video conferencing sessions: These sessions allowed the NP case managers to meet once a week as a group and with a cardiologist and the specialist NPs from the referral center to discuss problems regarding individual patients or problems with the case management program in their center.

5. Provision of telemedicine technology: Telemedicine technology was available for real-time consultation between the NP case managers and the referral center, either using video, peripheral monitoring, or telephone technology.

6. Provision of patient education documentation: Patient education was emphasized strongly as an important aspect of care provided by the NP case managers. Patient education materials were provided to the NPs, who were trained on patient education and the distribution of education materials to patients and families.

7. Provision of laptop computers: Laptop computers were provided to the NPs to facilitate documentation of and access to patient information, especially when the NPs were consulted about particular patients at home during off hours.

8. Provision of case manager training: The NP case managers attended an initial training session on how to manage CHF patients. The training was developed to enrich the nurse practitioners' existing knowledge of CHF pathophysiology, symptomatology, and identification of high-risk patients; to teach them how to utilize the CHF clinical guidelines, medication guidelines, and patient education materials; and to provide instruction on how to motivate patients and coordinate care with other healthcare professionals.

#### Rating of intervention components

The second step of the analysis involved following Miles and Huberman's recommendations for organizing and evaluating qualitative data that relate to a given construct [19]. First, textual material from the interview transcripts (phrases and sentences) for each component that reflected the extent of each participant's satisfaction with the individual intervention components and their perceptions of the quality and consistency of the use of these intervention components at their facility was entered into a matrix arranged by the eight components. One matrix was created for each participant. Initially, two of the authors (FPH and REK) coded text (*i.e.*, organized data into the matrix) from five interview transcripts (approximately 25% of our data). This coding was based on three categories of FOI developed by Klein and colleagues (2001): participant's satisfaction with the intervention and the quality and consistency of their use of the intervention [18]. During this initial review, a codebook was designed to specify coding rules for satisfaction, quality, and consistency relevant to each component (see Additional file 1, Codebook). One author (REK) coded text from the remaining 13 transcripts and assigned tentative ratings of FOI to each component by participant.

When this initial rating process was completed, four of the authors (REK, FPH, US, and JCL) reviewed and discussed the resultant matrices, made additional coding changes, and agreed on the final categorizations of the interview data. These authors also reviewed and discussed the matrices and FOI ratings and clarified the rating criteria by comparing and contrasting FOI ratings across the sample of transcripts. As a result of these consensus discussions, the three-category FOI scale was amended to a five-category scale to include the categories of low compliance and high compliance. The authors agreed that the five categories of FOI were appropriate, and definitions for rating criteria were determined (see Additional file 2, Definitions of Codes for Commitment to Use). Differences in opinions were discussed until full agreement between the four authors was achieved on the coding of the textual material from the participants and the FOI ratings for each component.

Four authors were included in the group consensus discussions to achieve a negotiated validity, a process in which interpretation of the data may vary with the orientation of each author [20]. Two of the authors (FPH and JCL) were involved in the implementation and evaluation of the intervention, one of the authors (US, a physician) provided clinical expertise, and the fourth (REK, primary) author served as the qualitative-methods expert. During group consensus discussions, the two authors with knowledge of the intervention offered explanations of how well the indications of use revealed in the interviews aligned with the intended intervention design. The other two authors provided objective input based on their clinical and methodological perspectives. This level of understanding is necessary to truly determine FOI as opposed to simply achieving reliability in coding and analysis, where a different group of researchers would produce the same results [21].

#### Analysis of medical center ratings

To determine an overall medical center FOI rating for each component, a meta-matrix was developed to display the FOI rating by participant (see Additional file 3, FOI Rating Matrix: Participant FOI Rating by Program Component). The overall medical center FOI ratings for each component were determined by group consensus discussions among the four authors, based on a review of the component FOI ratings across participants. During these discussions, the group consulted summaries prepared by REK of the rationale (including coded transcript excerpts) for each component's rating in the matrix.

After the FOI ratings were finalized for each individual component for each medical center, an overall FOI rating for each center was calculated by taking the average of all of the individual component ratings. However, we were primarily interested in the relative rankings of the four medical centers, rather than attributing a specific meaning to individual facility ratings of FOI. Therefore, after calculating medical center level FOI ratings from the average rating across components, we assigned a rank (1 to 4) to each center based on the FOI ratings.

### Data analysis: quantitative

The inpatient resource utilization outcomes (CHF hospital admissions, CHF hospital bed days of care, all-cause hospital admissions, and all-cause hospital bed days of care) were measured as counts for each patient in the sample and were included as the dependent variables in our negative binomial regression models. Covariates included age, race, number of comorbidities, medical center type (primary vs. tertiary), and the baseline values of the outcome variables one year prior to patient enrollment in the study. Mortality was modelled using Cox proportional hazards survival models. Deaths were included for patients who died between baseline and the end of the first year of the intervention. Similar to the resource utilization models, covariates included age, race, number of comorbidities, medical center type (primary vs. tertiary), and number of all-cause admissions in the year prior to enrollment. All analyses controlled for clustering within each medical center.

To determine the effect of FOI on outcomes, separate regressions were run for each of the outcome measures. FOI rank was entered into the models as a dummy variable, with the lowest FOI ranking (rank = 1) serving as the reference group in each model. This approach is preferable to a model in which rank is entered as a single variable with ordinal values (*i.e.*, 1, 2, 3, or 4) because such a variable assumes that the distance between the values is equivalent. As noted earlier, because we are not interested in attributing a meaning to the individual values of FOI, and are instead interested in the relative rankings of the medical centers, it was not appropriate to assume an equal distance between each of the FOI values. Analyses were performed using Stata version 11.1 (StataCorp LP, College Station, TX, USA).

## Results

### Research question 1: what is the FOI for each component of the intervention in each medical center?

The FOI ratings for each component by medical center are shown in Table 1. A summary of the qualitative findings supporting the ratings for each of the components is presented below. The ratings for components 1, 2, and 3 were based on interview responses from all participants. For the other components (4 through 8), the NPs were the only participants who made substantial comments about the component, so the medical center ratings for each of these components was based only on NP responses. Results for each component are as follows:

**Table 1:** Fidelity of implementation (FOI) ratings from qualitative ratings

Facility	**FOI Ratings**^**a **^**for Program Components**								**Average**^**b**^ FOI	FOI rank
	**1**^**c**^	**2**	**3**	**4**	**5**	**6**	**7**	**8**		

**A**	4	3	--	3	1	3	5	5	3.4	2

**B**	4	4	4	5	3	--	3	2	3.6	4

**C**	3	3	4	4	1	5	3	5	3.5	3

**D**	3	3	3	4	1	--	5	2	3.0	1

**Variance**	0.33	0.25	0.33	0.67	1.00	2.00	1.33	3.00		

Blank cells: ---, indicates missing data

^a^Ratings: 1 = nonuse, 2 = low compliance, 3 = compliant, 4 = high compliance, 5 = committed; ^b^total FOI rating was calculated by summing ratings across components and dividing by the number of components for which ratings were made; ^c^components: 1 = availability of a nurse practitioner (NP) case manager, 2 = collaboration between primary care providers and NP case managers, 3 = coordination between primary care (referring) centers and inpatient (referral) centers, 4 = provision of video conferencing sessions, 5 = provision of telemedicine technology, 6 = provision of patient education documentation, 7 = provision of laptop computers, 8 = provision of case manager training.

-- indicates missing data.

1. Availability of an NP case manager: The availability of the NP was a factor in the different medical center level FOI ratings for this component, as was satisfaction with the NP. Medical centers A and B both received a rating of high compliance for this component, mainly determined from the cardiologist's and PCPs' positive comments about the NP as an individual, the quality of care provided, and her independence of practice.

2. Collaboration between PCPs and NP case managers: The providers in medical center B were rated as committed to use based on their positive statements of consistently referring patients and the quality of communication with the NP. Medical centers A, C, and D received a rating of compliant for component 2 because participants made more negative and incongruous statements regarding the quality of communication and consistency of referrals between PCPs and the NP, reflecting inconsistencies and some frustration. Some problems with communication are illustrated by the following quote from a PCP in medical center D:

See the problem with the independent practitioners, some of the consults we have to see the response in the alert thing every time they see the patient, some we don't. It's variable. Sometimes we see the response right away, sometimes you don't see the response until you see the patient again and you look through it... I think it's a big problem.

The NP in medical center A also expressed frustration with communication:

...it's nice that we give reports on these people but it would be nice if we could also get reports on these patients when they [the PCPs] see them too. That would make it two-way communication and that doesn't always happen here.

Inconsistency in perceptions of the referral process also appears to be an issue among organizational members during the implementation of the intervention. For example, a PCP from medical center D noted that "anytime a patient is diagnosed we're encouraged to refer them right away," while a cardiologist from the same center stated:

...it's not about referral... it's basically when they come in here for the inpatient and they get admitted as heart failure patient, on the floor the research coordinator will track all these patients and if they're willing they get referred to the clinic... So the physicians at [facility D] never refer patients to [the NP].

3. Coordination between primary care (referring) centers and inpatient (referral) center: Medical center A, as the referral center, was not rated for this component. The NPs in medical centers B, C, and D all expressed satisfaction with the referral process and their ability to coordinate patient care; however, medical center D was rated lower than were medical centers B and C because the cardiologist said that referrals of CHF patients to the inpatient center did not occur, consequently contradicting the nurse practitioner's perception of the same facility indicating that these referrals did take place.

4. Provision of video conferencing sessions: This component was viewed positively among all NPs, with the exception of one NP in medical center A. Thus, medical center A was rated only as compliant because the NP expressed dissatisfaction with the sessions based on her belief that they were unnecessary for *her *because she worked in the same medical center as, and had direct access to, the cardiologist who led the sessions. In contrast, the other NP in medical center A expressed ownership over the sessions and a high level of satisfaction. The NPs from the other medical centers expressed a high level of satisfaction with the video conferencing sessions, in terms of the quality of the content and the regular opportunities to have their questions answered by the cardiologist. With the exception of the NP in medical center B, all of the NPs stated frustration with the unreliable video conferencing equipment. Thus, medical center B earned a ranking of committed, while C and D were rated as high compliance.

5. Provision of telemedicine technology: The NPs either had not received the telemedicine equipment or did not think the equipment was working in their medical centers, nor was a protocol for the use of the equipment established. Despite these problems, the NPs in medical centers A, C, and D expressed indifference with not being able to use the telemedicine equipment; they did not view this as a critical component of the program. Medical center B did not have the telemedicine equipment set up, but the NP consistently used the video conferencing equipment as a substitute to hold comparable telemedicine consultations with patients and the cardiologist at the referral center. The NP in medical center B expressed satisfaction with the cardiologist consultations that were supported by the telemedicine component. Thus, where medical centers A, C, and D were rated as nonuse, medical center B was rated as compliant.

6. Provision of patient education documentation: In the medical centers in which patient education documentation was discussed by the NPs (A and C), the differences in FOI ratings were based on NP perceptions of document availability. The quality of the content and patient receptivity to the education materials were not discussed in the interviews. In medical center A (rated as compliant), the NP had trouble obtaining the patient education documents; she was personally committed to obtaining education materials for patients but expressed frustration with not having enough assistance in obtaining needed materials. Medical center C received a rating of committed because the NP stated that the facility had patient education documentation for every CHF patient.

7. Provision of laptop computers: The two medical centers with committed ratings for this component were those in which the NPs stated satisfaction with the benefits of having increased access to patient information, so they could be responsive to patients during off hours. The two medical centers with compliant ratings had NPs who stated that they were already using laptops at home and had access to patient information but did not expand on any benefits from having more access to patient information.

8. Provision of case manager training: In the two medical centers that received a rating of committed, the NPs expressed that they felt they received an appropriate level of training. In the two medical centers that received a rating of low compliance, both NPs responded that the initial training was not adequate, and they wished they had had more training.

### Research question 2: what is the FOI for the intervention as a whole in each medical center?

#### Ranking by average FOI across all program components

To determine the FOI of the intervention as a whole, we combined the FOI ratings of the individual components, as shown in Table 1.

### Research question 3: how is FOI associated with intervention effectiveness?

A total of 457 patients from the four medical centers participated in the intervention. Table 2 shows baseline patient characteristics and inpatient resource utilization, as well as inpatient resource utilization and mortality after one year. At baseline, the CHF patient population at each medical center had some significant differences in racial composition, number of comorbidities, and all-cause admissions and days of care. At one year, significant differences were observed across medical centers for all-cause admissions, CHF admissions, and CHF days of care.

**Table 2:** Patient characteristics and resource utilization^a^

Patient characteristics	Facility A (N = 189)	Facility B (N = 44)	Facility C (N = 82)	Facility D (N = 142)	*p *value
Age	65.1 (10.1)	68.2 (10.2)	65.8 (10.6)	64.9 (11.3)	.3661

Other race besides white, frequency (%)	19 (12%)	3 (8%)	9 (14%)	83 (66%)	< .0001*

Comorbidities	3.7 (1.9)	3.3 (1.4)	3.3 (1.9)	2.1 (1.8)	< .0001*

**Baseline patient resource utilization**					

All-cause hospital admissions	1.2 (1.5)	0.5 (0.8)	1.4 (1.8)	0.9 (1.2)	.0011*

CHF hospital admissions	0.3 (0.6)	0.2 (0.5)	0.4 (0.8)	0.2 (0.6)	.1367

All-cause hospital days of care	6.8 (11.1)	4.7 (8.6)	8.3 (13.5)	4.5 (8.2)	.0408*

CHF hospital days of care	2.2 (7.8)	0.9 (2.6)	1.8 (4.1)	1.2 (4.1)	.4424

**Year 1 inpatient resource utilization**					

All-cause hospital admissions	0.9 (1.4)	0.8 (1.4)	0.5 (0.8)	0.5 (1.1)	.0080*

CHF hospital admissions	0.2 (0.5)	0.2 (0.8)	0.04 (0.2)	0.1 (0.4)	.0977

All-cause hospital days of care	5.4 (10.9)	3.8 (8.2)	2.1 (5.3)	2.6 (7.3)	.0087*

CHF hospital days of care	1.2 (4.2)	1.2 (5.4)	0.1 (1.6)	0.4 (0.8)	.0275*

**Year 1 mortality**					

Mortality, frequency (%)	18 (10%)	5 (11%)	11 (8%)	6 (7%)	.8240

^a^Data are presented as mean (SD) unless otherwise specified.

* *p *< .05.

CHF = chronic heart failure.

Table 3 shows the results of the regression models analyzing the effect of FOI rank on the five measures of intervention effectiveness. Criteria for exclusion from the models included missing patient values for the control variables age and race. (All other variables had complete data.) The final sample for the analyses comprised 387 patients. Significant effects of FOI rank (higher FOI rank associated with lower use of services and mortality compared to FOI rank 1) were observed for FOI rank 3 in all of the models. Neither FOI rank 2 nor FOI rank 4 was associated with lower use of services and mortality compared to FOI rank 1. Thus, the effect of FOI rank on patient outcomes was not as expected; the highest FOI rank (4) was not significantly associated with lower use of services and mortality compared to FOI rank 1.

**Table 3:** Significance of fidelity of implementation (FOI) rank (based on average FOI for eight intervention components) in predicting improved patient outcomes

	FOI rank (facility)		
**Patient outcomes at year 1**	**2** **(A)**	**3** **(C)**	**4** **(B)**

All-cause hospital admissions	.71	< .001*	.26

CHF hospital admissions	.72	< .05*	.57

All-cause hospital days of care	.33	< .001*	.27

CHF hospital days of care	.20	< .01*	.85

Mortality	.67	< .05^a^*	.16

^a^FOI was not significant for the first five months (*p *= .40), but after that, FOI = 3 predicted better survival (*p *< .05).

* *p *< .05.

CHF = chronic heart failure.

One of the possible reasons for observing the expected relationship between FOI rank 3 and FOI rank 1, but not between FOI rank 4 and FOI rank 1, is that the FOI ratings from which rank was calculated are so similar to each other, they lack variation, especially for medical centers A, B, and C. The FOI rating as calculated might not be doing a very good job of distinguishing among the medical centers in terms of the extent to which they implemented the intervention as intended. As part of a *post hoc *analysis, therefore, we recalculated FOI for each medical center based on those components of the intervention that varied the most in terms of their implementation across the medical centers. An examination of the variance in FOI ratings for each intervention component (bottom row, Table 1) shows that components 1, 2, and 3 were implemented very consistently across medical centers; in contrast, components 4 to 8 were more variable (variance ≥0.67) in their implementation. Table 4 shows the revised FOI ranks for each of the medical centers based on the average FOI ratings calculated from the five intervention components 4 to 8.

**Table 4:** FOI rank based on average FOI rating for five intervention components with variance ≥0.67

Facility	Average FOI rating	FOI rank
A	3.40	3

B	3.25	2

C	3.60	4

D	3.00	1

FOI = fidelity of implementation.

Table 5 shows the results of the regression models analyzing the effect of FOI rank on patient outcomes based on the revised calculation of FOI rating. Significant effects of FOI rank (higher FOI rank associated with lower use of services and mortality compared to FOI rank 1) were observed for FOI rank 4 in all of the models. A review of the adjusted means for each of the patient outcomes from these models (not shown) reveals that while the medical center with FOI rank 4 has significantly better outcomes (lower inpatient resource utilization and mortality) than the facility with FOI rank 1, medical centers with FOI ranks of 2 and 3 have the worst outcomes. Thus, it appears that the medical centers with the highest FOI, rank = 4, has predictive validity relative to FOI ranks 1, 2, or 3, but an FOI rank of 2 or 3 is not associated with better outcomes compared to FOI rank 1.

**Table 5:** Significance of FOI rank (based on average FOI for five intervention components with variance ≥0.67) in predicting improved patient outcomes

	FOI rank (facility)		
**Patient outcomes at year 1**	**2** **(B)**	**3** **(A)**	**4** **(C)**

All-cause hospital admissions	0.26	0.71	< 0.001*

CHF hospital admissions	0.57	0.72	< 0.05*

All-cause hospital days of care	0.27	0.33	< 0.001*

CHF hospital days of care	0.85	0.20	< 0.01*

Mortality	0.16	0.67	< 0.05^a^*

^a^FOI was not significant for the first five months (*p *= .40), but after that, FOI = 4 predicted better survival (*p *< .05).

* *p *< .05.

FOI = fidelity of implementation; CHF = chronic heart failure.

## Discussion

The need to further understand FOI has been identified in the case management literature [22], the healthcare literature [1], the social sciences literature [6], and the implementation science literature [23]. The literature identifies this gap in understanding as being two-fold--defining the concepts to be measured and also developing measures that can be used for assessing FOI for distinctly different interventions [3]. The methods described in this paper can help address these gaps in measuring and understanding FOI. We developed a generalizable method for measuring FOI that is adaptable to the specific intervention using component analysis. We define component analysis as the process of assessing the individual intervention components as a means of determining the extent to which an intervention is implemented as intended. This type of method is supported in the sentiments of implementation scholars calling for future empirical analysis to use a more systematic method of examining complex interventions [24]. Our methods offer a systematic means of examining the dimensions of FOI for each intervention component, then quantifying those data to allow for an examination of the correlation of FOI with patient outcomes. We have put forth qualitative findings that demonstrate the adaptability of the dimensions of FOI--consistency, quality, and satisfaction--in assessing the level of FOI for different organizational members or users of the intervention components.

We also put forth an approach to test the predictive validity of the FOI construct association with intervention effectiveness. Predictive validity assesses the ability of the operationalization of a construct to predict something it should theoretically be able to predict [25]. Our conceptual framework posits that a facility's level of FOI will be associated with intervention effectiveness--defined as improved patient outcomes (decreased resource utilization and mortality). We examined the effect of medical center FOI rankings on patient outcomes, where the rankings were based on two different calculations of FOI. Those rankings based on FOI calculations that included intervention components implemented variably across the four participating medical centers showed better predictive validity than rankings based on all eight intervention components.

The components that were implemented variably across the medical centers included components 4 (provision of video conferencing sessions), 5 (provision of telemedicine technology), 6 (provision of patient education documentation), 7 (provision of laptop computers), and 8 (provision of training). Interestingly, components 1 (availability of an NP case manager) and 2 (collaboration between PCPs and NP case managers) were core components of the intervention and, not surprisingly, were implemented relatively consistently across medical centers. However, because of their variable implementation, the other components had a greater impact on intervention outcomes.

Our FOI measure based on the variably implemented components of the intervention shows promise in helping to understand the differences in outcomes observed among the medical centers; that is, the medical center with the best FOI ratings for components 4 to 8 had the best patient outcomes. However, medical centers with intermediate FOI ratings did not have better outcomes than the medical center with the lowest FOI ratings, suggesting that our measure of FOI is not completely valid. Nevertheless, our approach to calculating and analyzing FOI can hopefully serve as an example of a way in which mixed methods can be used to understand the role of FOI in implementation research. Future research can improve on the limitations described below.

## Limitations

Our study has several limitations. First, the participant interviews did not specifically target all three dimensions of FOI (*i.e.*, consistency, satisfaction, and quality of use); the interview guide used to collect the qualitative data was designed primarily to assess aspects of organizational member satisfaction and commitment to the intervention. In addition, although questions were comparable, different organizational members were asked somewhat different questions about the intervention and were not asked about each component specifically. A second limitation is our inability to measure all differences between the four medical centers that might influence patient outcomes. We tried to include variables that we believe captured the most important differences--*i.e.*, patient characteristics (measured by baseline utilization), primary versus tertiary facility, and FOI. Nevertheless, there may have been other important organizational variables that we did not identify (*e.g.*, leadership support, organizational culture, tension for change, incentives, and rewards), emphasizing the importance for their measurement as indicators of organizational context in future research [26]. Third, there are potential limitations in the generalizability of the research context. The four medical centers operated within the same integrated delivery system. The elements of good chronic illness care are likely less difficult to implement in integrated delivery systems such as the VA, which has a defined population, comprehensive services, a preventative orientation, and a standardized electronic medical record [27].

## Conclusions

FOI is an important but complex phenomenon that can be difficult to measure. The results of this analysis have revealed important considerations for measuring and analyzing FOI in an effort to understand the translation of evidence-based care into clinical practice. The method of component analysis brought forth the importance of assessing the FOI of each component of a complex intervention based on multiple organizational members' perceptions and identifying those components that were implemented inconsistently across sites. The mixed-methods approach allowed us to correlate the medical center with the highest FOI rank based on these components with the best patient outcomes. This specific finding provides an important message to clinicians and administrators interested in implementing a similar CHF case management program; all of the program components should be implemented in a manner promoting consistency, satisfaction, and quality of use. The more general finding--that a measure of FOI with some predictive validity can be calculated using component analysis and a mixed-methods approach--can be useful to implementation researchers who need to consider FOI as an important factor in understanding potential differences across organizations in achieving the desired outcomes from implementing an intervention.

## Competing interests

The authors declare that they have no competing interests.

## Authors' contributions

REK and JCL conceived of the study. REK, FPH, US, WW, and JCL participated in the design and analyses. REK and JCL led the writing, and all authors read and commented on drafts and approved the final manuscript.

## Supplementary Material

Additional file 1 **Codebook**. Click here for file

Additional file 2 **Definitions of Codes for Commitment to Use**. Click here for file

Additional file 3 **FOI Rating Meta-Matrix: Participant FOI Rating by Program Component**. Click here for file
