# Supplementary material for: Fidelity of implementation: development and testing of a measure
Source: Implement Sci. 2010 Dec 30;5:99. doi: 10.1186/1748-5908-5-99 (PMC3161382; doi:10.1186/1748-5908-5-99)
Supplement: Additional file 1 — Codebook. [file 1748-5908-5-99-S1.PDF]

Appendix A. Codebook

1. **Nurse Practitioner Case Manager**		
**Indication of Use**	**Definition**	**Coding Rules**<br>(categorized by interview participant)
**Satisfaction**<br>(Independence)	The provider's perception of the NP case manager overall, as well as the level of independence with which the NP managed CHF patients' cardiac care and medical needs. This code includes an assessment of the NP's ability to practice independent of a physician's oversight, to consult with a physician when necessary, and to provide expertise to assist PCPs in the management of CHF patients.	*Cardiologist & Primary Care Provider (PCP)*: code statements regarding the NP's ability to practice independently. Also code statements indicative of provider's perception of the NP case manager overall.<br><br>*Nurse Practitioner (NP)*: code statements regarding limitations of the facility in supporting the NP in carrying out the role of case manager (i.e. too much independence and not enough administrative support).
**Consistency**<br>(Availability)	The available time the NP had to accept CHF patient referrals from PCPs and whether or not the NP had enough time to treat CHF patients and consult with PCPs regarding their CHF patients.	*Cardiologist, PCP, & NP:* code statements regarding the provider's perception of the NP having sufficient time to see CHF patients and to take care of their needs in regards to CHF care.
**Quality**<br>(Quality of Care)	The quality, patient satisfaction, or cost effectiveness associated with the care provided by the NP as a result of the NP case management program.	*Cardiologist, PCP, & NP:* code statements regarding quality of care, patient satisfaction, or cost effectiveness associated with the NP case manager.

2. Collaboration between Primary Care Physicians and Nurse Practitioner Case Managers		
**Indication of Use**	**Definition**	**Coding Rules**<br>(categorized by interview participant)
**Satisfaction**	Enthusiasm or frustration with the collaboration between the PCP and NP.	*Cardiologist & PCP:* code positive or negative statements made about collaboration with the NP.<br><br>*NP:* code positive or negative statements made about collaboration with the PCP.
**Consistency**<br>(Frequency)	The rate at which PCPs referred CHF patients to the NP. This code also captures if PCPs did not refer CHF patients to the NP.	*Cardiologist, PCP*, & *NP*: code statements indicative of how often providers referred CHF patients to the NP.
**Quality**<br>(Quality of Communication & Use of Information)	Assessment of the means of communication, communication channels, and ability to communication when necessary. This code only captures communication specific to CHF case management and not communication between PCPs and NPs in general.	*Cardiologist, PCP, & NP:* code for quality, timeliness, and completeness of communication between the PCP and the NP. Also code examples of communication between the PCP and the NP.

**3. Coordination between Primary Care (Referring) Centers and Inpatient (Referral) Facility**		
**Indication of Use**	**Definition**	**Coding Rules**<br>(categorized by interview participant)
**Satisfaction**	Enthusiasm or frustration with the referrals between the referring center and the referral facility.	*Cardiologist, PCP, & NP:* code positive or negative statements made about the process of referring patients to the referral facility.
**Consistency**<br>(Frequency)	The rate at which the referring facilities referred CHF patients to the referral facility. This code also captures if the referral did not take place.	*Cardiologist, PCP, & NP*: code statements indicative of the occurrence referral of patients to facility A for CHF related procedures.<br>Note: Not all cardiac procedures are related to CHF.
**Quality**<br>(Access & Quality of Communication)	Access – Whether the referring facilities were able to refer patients to the referral facility and have them admitted into the hospital.<br>Communication – Assessment of the means of communication, communication channels, and ability to communicate when necessary regarding referrals made to the hospital.	*Cardiologist, PCP, & NP:*<br>Access: code for ease or difficulty of getting patients admitted into hospital.<br>Communication: code for the quality, timeliness, and completeness of communication between referring and referral facilities.<br><br>Note 1: Facility A is the referral facility. Unless facility A participants made statements regarding the referrals they received from referring sites, data will be “missing” for facility A.<br><br>Note 2: This code only captures referrals specific to CHF care and not cardiology referrals in general.

**4. Video Conference Sessions**		
**Indication of Use**	**Definition**	**Coding Rules**
**Satisfaction**	Enthusiasm or frustration with the video conference sessions.	*NP Only:* code statements indicative of NP’s perception of the video conference sessions (i.e. whether or not the NP found the video conference sessions helpful for CHF care.)
**Consistency**<br>(Frequency)	Whether the video conference sessions took place on a regular basis.	*NP Only:* code statements regarding whether or not the video conference sessions took place every week, as planned.
**Quality**<br>(Access)	Whether all facilities were able to connect for the video conference sessions.	*NP Only:* code statements regarding barriers or facilitators to the participants connecting via video conference technology.

5. Telemedicine Technology		
**Indication of Use**	**Definition**	**Coding Rules**
**Satisfaction**	Enthusiasm for or frustration with the telemedicine technology.	*NP Only:* code statements indicative of NP's perception of the telemedicine technology.
**Consistency** (Frequency)	Whether the telemedicine consults occurred on a regular basis.	*NP Only:* coding is straight forward based on the interview questions and responses.
**Quality** (Access)	Whether the NPs were able to connect with a cardiologist and use the telemedicine equipment for telemedicine consultations.	*NP Only:* code statements regarding barriers and facilitators to using the telemedicine technology.

6. Patient Education Documentation		
**Indication of Use**	**Definition**	**Coding Rules**
**Satisfaction**	Enthusiasm for or frustration with the patient education documentation.	*NP Only:* code statements indicative of NP's perception of the patient education documentation (i.e. whether or not the NP finds the patient education documentation helpful for CHF care).
**Consistency** (Availability)	Whether the facility provided the NP with patient education documentation to give to the CHF patients, and whether the NP gave the documentation to CHF patients.	*NP Only:* code statements regarding whether or not the NP distributed patient education documentation to CHF patients.
**Quality**	General quality of the patient education documentation used by the NP.	*NP Only:* code statements regarding barriers or facilitators to using patient education documentation.

7. Laptop Computers		
**Indication of Use**	**Definition**	**Coding Rules**
**Satisfaction**	Enthusiasm for or frustration with the laptop computers.	*NP Only:* code statements indicative of the NP's perception of the laptop computers (i.e. whether or not the NP finds the laptop computers helpful for CHF care.)
**Consistency** (Availability)	Whether the NP received a laptop computer and the amount of use of the laptop expressed by the NP.	*NP Only:* code statements regarding how often the NP used the laptop computers for CHF care.
**Quality**	Quality of use was not assessed for this component.	

**8. Training**		
**Indication of Use**	**Definition**	**Coding Rules**
**Satisfaction**	Enthusiasm for or frustration with the training.	*NP Only:* code statements indicative of the NP's perception of the training (i.e. whether or not the NP found the training helpful for CHF care).
**Consistency** Occurrence	Whether the NP received the training that was intended by the program planners.	*NP Only:* code statements indicative of the extent of NP training for the CHF NP case management program.
Quality	General quality of the training in preparing the NP to treat and manage CHF patients, and generally fulfill their role in the program, as a nurse practitioner case manager for CHF patients.	*NP Only:* code statements regarding the adequacy of the training as perceived by the NP in handling the problems encountered with CHF patients.
