# Supplementary material for: Fidelity of implementation: development and testing of a measure
Source: Implement Sci. 2010 Dec 30;5:99. doi: 10.1186/1748-5908-5-99 (PMC3161382; doi:10.1186/1748-5908-5-99)
Supplement: Additional file 2 — Definitions of Codes for Commitment to Use. [file 1748-5908-5-99-S2.PDF]

Appendix B. Definitions of Codes for Commitment to Use

(1) Non-use: If participant states explicitly that they did not use the component. Example: When asked about referring patients to the NP for CHF, and the physician responded that he/she did not refer patients to the NP.

(2) Low-compliance: If participant discusses using the component and speaks negatively in reference to the component, or if participant speaks positively about the component but mentions barriers to using or does not use the component according to the protocol.
Example: When asked what tasks he felt the NP case manager was best suited for, the provider responded, "Ordering that medications that I am not permitted to order." He was then asked a follow-up question about any other tasks the NP case manager was suited for that he hadn't used the NP for, and he responded "Nope."

(3) Compliant Use: If participant discusses using the component according to protocol and: (a) speaks both positively or negatively about the component; or (b) does not speak either positively or negatively about the component; or (c) discusses barriers to using the component appropriately while speaking positively of the component.
Example: One NP said that she used the patient education materials, but when asked if there was anything she would like more assistance with, she responded:

> "I would like more assistance with obtaining teaching materials and handouts. Right now we have some old copies that we keep copying over and over. And we have to copy them. And then we have to order them; we have booklets that we order. It would be nice to turn that over to a secretary and say "Hey, we're running low on this handout, or this booklet. Could you please get those for us? And could you also do the packets for us so that when we get a new patient we have their packet all ready to go?" We could just give it to the patient and review it, as opposed to getting packets together and making sure that we have all of our handouts printed and making sure that we're not using the last handout, that type of thing."

(4) High Compliance: If participant discusses using the component according to protocol and speaks positively in reference to the component, but also has a suggestion for improvement. This rating allows giving a rating to nuanced responses.
Example: When asked about the timeliness of obtaining information from NP case managers about patients, the provider responded by underscoring that information exchange is important; however, he also had suggestions for improvement:

> "…I'd have to give that a little bit lower score than the other comments. I think with acute issues we have a very good mechanism right now for that transfer of information. …the routine quarterly reports of the progress of the patient may not be necessary. Basically, if the patient's doing well, I don't need to know about it. But if there is a failure in the patient's improvement, they're becoming a frequent user of hospital resources, a very prompt notification would be more important. SO I would focus the

> energies of the nurse practitioner on when to contact the primary care doctor and say that they should continue to monitor this population.”

(5) Committed Use: If participant states explicitly that they use the component according to protocol and speaks positively in reference to the component without qualifications.
Example: When asked about referring patients to the NP case manager providers responded:

> “… she does an excellent job and I think she’s been very instrumental in providing good care to heart failure patients… …both from an educational standpoint and from a management standpoint.”

> “I have a wonderful time with [the nurse practitioners]. I think that they’re very focused and they have no problems conveying important issues to me.”

(6) Missing: If participant does not discuss any aspect of the component during the interview.
