# Supplementary material for: Fidelity of implementation: development and testing of a measure
Source: Implement Sci. 2010 Dec 30;5:99. doi: 10.1186/1748-5908-5-99 (PMC3161382; doi:10.1186/1748-5908-5-99)
Supplement: Additional file 3 — FOI Rating Meta-Matrix: Participant FOI Rating by Program Component. [file 1748-5908-5-99-S3.PDF]

Appendix C. FOI Rating Matrix: Participant FOI Rating[1] by Program Component

Component 1, Nurse Practitioner Case Manager						
**Site**	**Cardiologist**	**PCP MD 1**	**PCP MD 2**	**PCP NP & Care Manager**	**NP (2 at Site A)**	**Facility Total**
**A**	Committed	Committed	Compliant	Compliant & High Compliance	Missing & Compliant	**High Compliance (4)**
**B**	no interview	Committed	Committed	no interview	Compliant	**High Compliance (4)**
**C**	Committed	Low Compliance	Compliant	no interview	Low Compliance	**Compliant (3)**
**D**	Committed	Compliant	Compliant	no interview	Compliant	**Compliant (3)**

Component 2, Collaboration between PCPs and NP Case Managers						
**Site**	**Cardiologist**	**PCP MD 1**	**PCP MD 2**	**PCP NP & Care Manager**	**NP (2 at Site A)**	**Facility Total**
**A**	High Compliance	Compliant	Compliant	Compliant & High Compliance	Compliant & High Compliance	**Compliant (3)**
**B**	no interview	Committed	Committed	no interview	Low Compliance	**High Compliance (4)**
**C**	Committed	Low Compliance	Compliant	no interview	Compliant	**Compliant (3)**
**D**	Compliant	Low Compliance	High Compliance	no interview	Compliant	**Compliant (3)**

Component 3, Referrals and Communication between Referring and Referrals Facilities						
**Site**	**Cardiologist**	**PCP MD 1**	**PCP MD 2**	**PCP NP & Care Manager**	**NP (2 at Site A)**	**Facility Total**
**A**	Missing	Missing	Missing	Missing & High Compliance	Non use & Missing	**Missing**
**B**	no interview	Compliant	High Compliance	no interview	High Compliance	**High Compliance (4)**
**C**	Missing	Missing	Missing	no interview	Committed	**High Compliance (4)**
**D**	Nonuse	Missing	Compliant	no interview	High Compliance	**Compliant (3)**

Component 4, Video Conference Sessions						
**Site**	**Cardiologist**	**PCP MD 1**	**PCP MD 2**	**PCP NP & Care Manager**	**NP (2 at Site A)**	**Facility Total**
**A**	N/A	N/A	N/A	N/A & N/A	Low Compliance & High Compliance	**Compliant (3)**
**B**	no interview	N/A	N/A	no interview	Committed	**Committed (5)**
**C**	N/A	N/A	N/A	no interview	High Compliance	**High Compliance (4)**
**D**	N/A	N/A	N/A	no interview	High Compliance	**High Compliance (4)**

Component 5, Telemedicine						
**Site**	**Cardiologist**	**PCP MD 1**	**PCP MD 2**	**PCP NP & Care Manager**	**NP (2 at Site A)**	**Facility Total**
**A**	N/A	N/A	N/A	N/A & N/A	Nonuse & Nonuse	**Nonuse (1)**
**B**	no interview	N/A	N/A	no interview	Compliant	**Compliant (3)**
**C**	N/A	N/A	N/A	no interview	Nonuse	**Nonuse (1)**
**D**	N/A	N/A	N/A	no interview	Nonuse	**Nonuse (1)**

Component 6, Patient Education Documentation						
**Site**	**Cardiologist**	**PCP MD 1**	**PCP MD 2**	**PCP NP & Care Manager**	**NP (2 at Site A)**	**Facility Total**
**A**	N/A	N/A	N/A	N/A & N/A	Compliant & Missing	**Compliant (3)**
**B**	no interview	N/A	N/A	no interview	Missing	**Missing**
**C**	N/A	N/A	N/A	no interview	Committed	**Committed (5)**
**D**	N/A	N/A	N/A	no interview	Missing	**Missing**

Component 7, Laptop Computers						
**Site**	**Cardiologist**	**PCP MD 1**	**PCP MD 2**	**PCP NP & Care Manager**	**NP (2 at Site A)**	**Facility Total**
**A**	N/A	N/A	N/A	N/A & N/A	Committed & Committed	**Committed (5)**
**B**	no interview	N/A	N/A	no interview	Compliant	**Compliant (3)**
**C**	N/A	N/A	N/A	no interview	Compliant	**Compliant (3)**
**D**	N/A	N/A	N/A	no interview	Committed	**Committed (5)**

Component 8, Training						
**Site**	**Cardiologist**	**PCP MD 1**	**PCP MD 2**	**PCP NP & Care Manager**	**NP (2 at Site A)**	**Facility Total**
**A**	Missing	Missing	Missing	Missing & Missing	Committed & Compliant	**Committed (5)**
**B**	no interview	Missing	Missing	no interview	Low Compliance	**Low Compliance (2)**
**C**	Missing	Missing	Missing	no interview	Committed	**Committed (5)**
**D**	Missing	Missing	Missing	no interview	Low Compliance	**Low Compliance (2)**

[1]Ratings: 1 = non-use, 2 = low compliance, 3 = compliant, 4 = high compliance, 5 = committed
